# Assessing the Impact of Imaging Parameters on MRI Measurement of Kidney T_2_

**DOI:** 10.1002/jmri.70127

**Published:** 2025-09-25

**Authors:** Alexander J. Daniel, Susan T. Francis

**Affiliations:** ^1^ Sir Peter Mansfield Imaging Centre University of Nottingham Nottingham UK; ^2^ NIHR Nottingham Biomedical Research Centre Nottingham University Hospitals NHS Trust and School of Medicine Nottingham UK

**Keywords:** kidney, quantification, standardization, T_2_ relaxation

## Abstract

**Background:**

T_2_‐mapping has promise to evaluate kidney pathophysiology. Prior studies show a large variance in kidney T_2_, likely due to the differing acquisition sequences.

**Purpose:**

To compare four T_2_‐mapping sequences to investigate kidney T_2_.

**Study Type:**

Phantom and prospective in vivo assessments.

**Participants:**

ISMRM/NIST and QASPER phantoms; 8 healthy volunteers (4 female, 30 ± 8 years).

**Field Strength/Sequence:**

3 T, spin echo‐echo planar imaging (SE‐EPI), multi‐echo‐spin echo (ME‐SE), Gradient and Spin Echo (GraSE) vendor‐provided sequences, and custom T_2_‐prepared EPI, Dual‐echo B_0_‐mapping and DREAM B_1_‐mapping.

**Assessment:**

T_2_‐mapping accuracy in the ISMRM/NIST phantom in the presence of B_0_ frequency offset and B_1_^+^ by scaling of flip angles, and in the QASPER phantom in the presence of diffusion by altering pump rate compared to being turned off. Participants underwent a single 45‐min exam to collect four T_2_‐mapping sequences, B_0_ and B_1_ maps. In vivo T_2_ values compared across sequences and the influence of B_0_ and B_1_^+^ was evaluated.

**Statistical Tests:**

Shapiro–Wilk, Wilcoxon signed‐rank, Student's *t*‐test, Coefficient of Variation, Pearson's correlation coefficient, linear mixed effect model. *p* < 0.05 considered statistically significant.

**Results:**

SE‐EPI, ME‐SE, GraSE, and T_2_‐prepared EPI had a mean absolute scaled error of 0.52, 0.52, 0.36, and 0.27 over the kidney T_2_ range of the ISMRM/NIST phantom. GraSE was most robust to perturbations in B_0_/B_1_^+^. In the QASPER phantom, SE‐EPI was highly sensitive to diffusion leading to T_2_ shortening (66%), while multi‐echo sequences had lower diffusion sensitivity ordered by shortest echo spacing (ME‐SE 81%, T_2_‐prepared EPI 90%, GraSE 95% reduction in T_2_). In vivo, SE‐EPI measured T_2_ was significantly lower than multi‐echo sequences, and SE‐EPI and T_2_‐preparation had a −0.52 ± 0.08 and −0.57 ± 0.06 ms/% dependence on B_1_^+^.

**Data Conclusion:**

To reduce B_0_, B_1_^+^, and diffusion sensitivity for kidney T_2_‐mapping, a multi‐echo sequence spanning echo times up to the kidney T_2_ (~140 ms at 3 T) is recommended. Collecting data with different echo spacings can isolate the diffusion‐related T_2_ component.

**Evidence Level:**

2.

**Technical Efficacy:**

Stage 1.

## Plain Language Summary

The kidneys are sensitive to inflammation and build‐up of fluid; T_2_‐mapping can measure these changes and aid studies in kidney disease. This paper investigates how the choice of T_2_‐sequence effects the measured T_2_ value of the kidney. T_2_‐mapping sequences were collected in reference objects (phantoms) and healthy volunteers to determine which gave the most accurate and reliable results. The study found some methods underestimate T_2_ due to high kidney blood flow, while others are very sensitive to magnetic field variations inside the scanner. The study recommends a T_2_‐mapping sequence with shorter data sampling gaps to reduce these issues.

## 1 Introduction

The kidneys are structurally and functionally complex, highly perfused organs [1]. Spin–spin (T_2_) relaxation time mapping allows quantification of tissue composition reflecting free water content and has been shown to be sensitive to tissue inflammation and edema [2]. Quantitative T_2_‐mapping was initially developed for musculoskeletal imaging, with usage including the differentiation of cartilage type [3]. Applications have since considerably expanded, with T_2_‐mapping now routinely used in cardiac MRI to assess myocardial edema [4] and iron overload [5]. In neuroimaging, it is used to characterize tumors [6] and in studies of multiple sclerosis [7], epilepsy [8], dementia [9], and Parkinson's disease [10], with typical gray and white matter T_2_ values at 3 T being 80–100 and 100–110 ms, respectively [7, 8, 9, 10]. T_2_‐mapping has also been shown to be sensitive to the degree of hypoxia and hyperoxia [11]. However, T_2_‐mapping has had limited application in renal MRI [12], with the PARENCHIMA consensus paper [13] highlighting key knowledge gaps and lack of agreement in kidney T_2_ measures.

Several T_2_‐mapping sequences are available including: (i) Spin Echo‐Echo Planar Imaging (SE‐EPI), (ii) Multi‐Echo‐Spin‐Echo (ME‐SE) or Multi‐Echo‐Fast Spin‐Echo (ME‐FSE) [14], (iii) Gradient Spin Echo (GraSE) [15], and (iv) T_2_‐preparation. Studies of T_2_‐mapping in healthy participants have shown considerable variation in measured kidney T_2_ [12], ranging in the cortex and medulla from 87 ms [16] to 130 ms [17] and 85 ms [16] to 143 ms [18] at 1.5 T, and from 76 ms [16] to 132 ms [19] and 59 ms [20] to 138 ms [21] at 3 T, likely due to the different T_2_‐mapping sequences and image parameters used (Table S1). The differing T_2_‐mapping sequences may also vary in their sensitivity to diffusion, which leads to an underestimation of T_2_ [22], important as the kidney is a highly perfused organ (~400 mL/100 g/min) [1] and comprises tubules which are convoluted in the cortex and straighter in the medulla.

In multi‐echo sequences [14, 15] (ME‐SE and GraSE), non‐ideal RF pulse profiles can decrease the first echo signal, while subsequent echo signals can be hyper‐intense due to superimposed stimulated echo (STE) signals, which artificially prolong the measured T_2_. These STE signals arise as after the 2nd RF‐pulse transverse magnetization partially resides aligned (“locked”) around the B_0_ direction along the longitudinal axis and recovers with T_1_ relaxation. This “stored” longitudinal signal is then flipped back into the transverse plane by the 3rd RF‐pulse rephasing to form an STE. Methods such as the extended phase graph (EPG) algorithm have been shown to correct such STE effects [23, 24].

T_2_‐prepared EPI mapping [25] using non‐slice selective RF pulses for T_2_‐preparation has been shown to limit the effects of diffusion, imperfect refocusing pulses, and inhomogeneous B_1_ but requires a long acquisition time due to the need to repeat the scan for each slice. Adiabatic RF pulses such as B_1_‐insensitive refocusing (BIREF0 [26]), modified B_1_‐insensitive rotation [27], and Silver‐Hoult have been shown to further improve diffusion insensitivity and reduce sensitivity to the B_0_ and B_1_^+^ field; however, these can lead to a bias in measured T_2_ [28]. Echo time correction can be applied to account for the number and width of the refocusing pulse, and T_1_ and T_2_ relaxation times of target tissue [29].

To date, the application of renal T_2_‐mapping in human studies has focused on the early diagnosis of Autosomal Dominant Polycystic Kidney Disease [17, 19] and the assessment of renal cell carcinoma [30] using ME‐SE and T_2_‐preparation [20, 31]. Additionally, allograft function has been assessed in mice using ME‐SE [32].

The aim of this study was to investigate the image parameters which influence kidney T_2_ measurements by comparing SE‐EPI, ME‐SE, GraSE, and T_2_‐preparation with EPI readout T_2_‐mapping sequences in healthy volunteers. A further aim was to evaluate the influence of B_0_ and B_1_^+^ fields and sensitivity to diffusion in phantoms for each sequence.

## 2 Materials and Methods

Data were acquired on a 3 T Ingenia system (Philips Healthcare, Best, the Netherlands; software version 5.6.1).

### 2.1 Pulse Sequences

**FIGURE 1:**
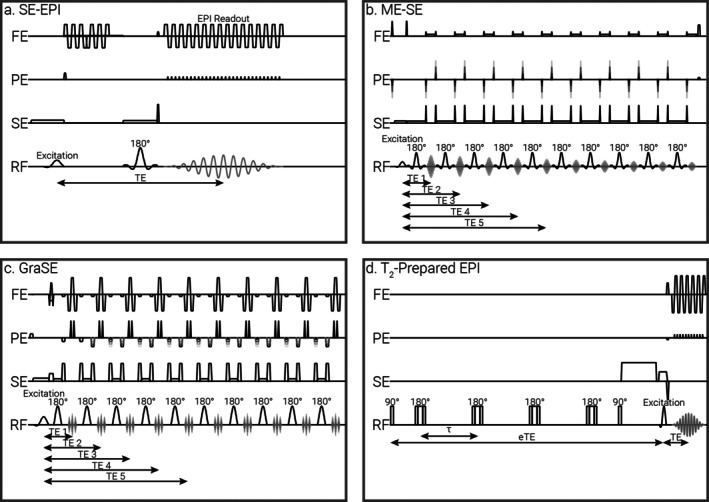
T_2_ mapping sequences of (a) SE‐EPI, (b) ME‐SE, (c) GraSE, and (d) T_2_‐prepared EPI used in this study. The 180° pulses in the T_2_‐prepared EPI are non‐selective; 180° pulses in the GraSE, ME‐SE, and SE‐EPI are selective and of 2× the thickness of the excitation slice. FE is the frequency encode direction, PE is the phase encode direction, and SE is the slice encode direction.

Figure 1 shows the renal T_2_‐mapping sequences evaluated in this study.

SE‐EPI T_2_‐mapping used a widely available vendor‐provided sequence. It comprises a 90° excitation pulse followed by a 180° RF pulse after time TE/2, with a single echo formed at time TE; here, this is sampled using an EPI readout. Repeats of the single echo SE‐EPI are then collected at different TEs to sample the T_2_ decay (Figure 1a).

ME‐SE T_2_‐mapping comprised a train of nominal 180° refocusing RF pulses with an echo forming between each. The same line of k‐space was then sampled at multiple echo times after a single excitation with this echo train repeated to sample each line of k‐space (Figure 1b). The ME‐SE was a vendor‐provided sequence with a commercial product key to allow the collection of > 8 echoes.

GraSE T_2_‐mapping combined a fast spin echo pulse sequence with EPI. A train of nominal 180° refocusing pulses generated a series of spin echoes, with gradient echoes sampled before and after each spin echo using an EPI readout and an effective echo time determined by the echo used to encode the central region of k‐space (Figure 1c). This was a vendor‐provided sequence; however, this sequence is not available on all MR vendors.

T_2_‐preparation T_2_‐mapping was collected with an EPI readout using a custom implementation. The T_2_‐preparation used a non‐selective 90° excitation to tip the magnetization into the transverse plane, with a train of 2–16 non‐selective refocusing pulses with Malcolm Levitt (MLEV) phase cycling [28, 33] used to achieve different effective echo times (eTE) (Figure 1d), followed by a non‐selective −90° pulse with a gradient to restore the T_2_‐weighted magnetization and crush any remaining transverse magnetization.

### 2.2 Data Acquisition

SE‐EPI, ME‐SE, GraSE, and T_2_‐prepared EPI sequences were collected with a spatial resolution and field of view (FoV) appropriate for kidney imaging, with acquisition parameters provided in Table 1. Dual‐echo gradient echo B_0_‐mapping (with ΔTE = 2.3 ms) and Dual Refocusing Echo Acquisition Mode (DREAM) B_1_‐mapping were collected with a matched FoV and spatial resolution. Data sets were collected using vendor‐provided B_0_ and B_1_ shimming in pencil‐beam volume and volume mode. For in vivo data, a shim box was placed over both kidneys. Respiratory triggering was used, acquiring T_2_‐mapping data at end‐expiration, as such acquisition times vary depending on participant breathing rate. The nominal acquisition time of scans was < 2 min 30 s to allow the sequence to be used within a multiparametric renal MRI protocol. To achieve this, the number of echo times and echo spacing was varied.

**TABLE 1:** Acquisition parameters for SE‐EPI, ME‐SE, GraSE, and T_2_‐prepared EPI sequences.

	T_2_ mapping sequence			
	SE‐EPI	ME‐SE	GraSE	T_2_‐prepared EPI
Software availability on Philips	Default	Default up to 8 echoes, > 8 commercial product	Default	Custom in‐house sequence
TE (min:step:max) (ms)	20:10:70	13:13:130	11.2:5.6:173.3	0:20:160
Echo spacing (ms)	N/A	13	5.6	10
Echoes sampled	6	10	30 (+1 start‐up echo)	9
Minimum TR (ms)^a^	5000	3000	3000	3000
Acquired/reconstructed voxel size (mm^3^)	3 × 3 × 5/3 × 3 × 5	3 × 3 × 5/3 × 3 × 5	3 × 3 × 5/3 × 3 × 5	3 × 5.65 × 5/3 × 3 × 5
Acquisition matrix	96 × 96 × 5	96 × 96 × 5	96 × 96 × 5	96 × 51 × 5
Signal averages	2	1	1	1
Shots/packages	2	1	2	1
Acquisition mode	Multi‐Slice	Multi‐Slice	Multi‐Slice	Multiple 2D
Bandwidth (Hz/pixel)	40 (Phase) 1787 (Freq)	180 (Phase) 180 (Freq)	405 (Phase) 2268 (Freq)	113 (Phase) 2845 (Freq)
SENSE	2.55	2.55	2.55	3
Partial Fourier fraction	0.838	1	1	0.706
Fast spin echo factor	N/A	10	30	N/A
EPI factor	37	N/A	3	17
Nominal acquisition time^a^	1 min 45 s	1 min 57 s	2 min 6 s	2 min 23 s
Specific absorption rate (SAR) (W/kg)	0.1	1.5	2.3	1.1

*Note*: All sequences have a field of view of 288 × 288 × 25 mm^3^, excitation and refocusing flip angles of 90° and 180°, respectively, and use Spectral Presaturation with Inversion Recovery (SPIR) fat suppression.

^a^ These sequences are respiratory triggered, so the time between repetitions is variable based on breathing rate; here the minimum time between repetitions is listed.

### 2.3 Phantom Measures

Phantom data was collected using a 32‐channel Philips head coil.

**FIGURE 2:**
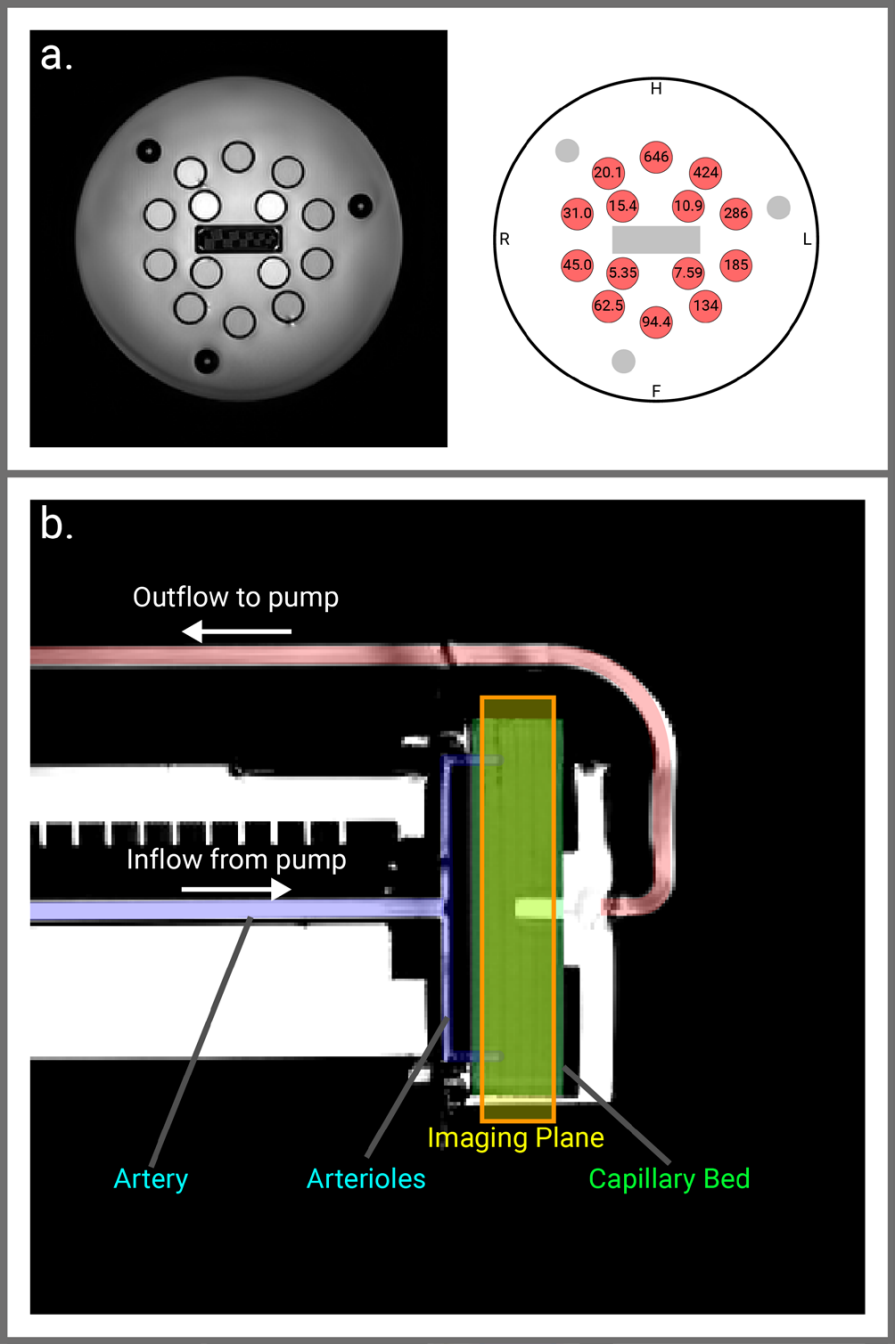
(a) ISMRM/NIST phantom showing T_2_ array and associated reference T_2_ values. (b) Schematic of QASPER phantom showing the flow of perfusate into a cylinder of porous media which simulates the “capillary bed,” and the location of the imaging plane used for T_2_ mapping measurements.

The ISMRM/NIST System Standard Model 130 (CaliberMRI, Boulder, CO, USA https://qmri.com) phantom T_2_ array comprised of 14 spheres with T_2_ of 5–650 ms at 3 T was used to assess the accuracy of T_2_ measurements against ground truth reference values, Figure 2a. This array was also used to evaluate the influence of B_0_ and B_1_^+^ field on T_2_ measurements. To modulate B_0_, the Philips “shimtool” was used to adjust the f_0_ offset from −200 to 200 Hz. To modulate B_1_^+^, each sequence was collected with all RF pulses scaled from 30% to 100% of the nominal flip angle. These percentages were then multiplied by the measured B_1_ map to determine the effective B_1_ (B_1eff_).

To assess the influence of diffusion on T_2_‐mapping, the QASPER (Quantitative Arterial Spin Labelling Perfusion Reference) perfusion phantom (Gold Standard Phantoms, https://goldstandardphantoms.com) composed of water‐based perfusate with NiCl added to modulate T_1_ to ~1.9 s at 3 T was used. Images of the phantom's simulated capillary bed (mean pore size 7 μm, porosity 32%) (Figure 2b) were collected for each T_2_‐mapping sequence with the pump producing continuous flow rates of 100–350 mL/min in 50 mL/min steps. The T_2_ at each flow rate was compared to the capillary bed with the pump turned off (with a measured T_2_ of 613 ± 19 ms for GraSE) to calculate the relative change in T_2_.

### 2.4 In Vivo Study

This study was approved by the local research ethics committee (H14082014), and healthy participants gave written, informed consent. Eight healthy participants confirmed to have no history of renal disease (4 female, age 30 ± 8 (range, 23–47) years, BMI 23.0 ± 2.3 (range, 26.1–19.4)) were scanned using a Philips 16‐channel anterior body coil array and the 16‐channel posterior array inbuilt to the scanner table. The in vivo renal MRI protocol was collected in a single 45‐min scan session and comprised a survey, localizers, the T_2_‐mapping sequences acquired in a fixed order (GraSE, ME‐SE, T_2_‐prepared EPI, and SE‐EPI), B_0_ and B_1_^+^ field maps, and T_2_‐weighted and T_1_‐weighted structural scans for segmentation of the whole kidney and cortex/medulla respectively [34]. Collection of sequences in a single session removed the consideration of physiological changes such as hydration status.

### 2.5 Data Analysis

Analysis was performed by two researchers (AD and SF, with 9 and 30 years MRI experience, respectively). T_2_‐mapping data were fit voxel‐wise to a mono‐exponential decay, $S\left(t\right)=S_{0}\cdot e^{-t/T_{2}}$ where $S\left(t\right)$ is measured signal at time t, $S_{0}$ is estimated signal at t=0, t is the time data is acquired (TE) and $T_{2}$ is measured T_2_, for all fits the *R* ^2^ was computed as the goodness of fit. Raw and fitted signals were plot against TE. This was implemented using Python 3.8 and the United Kingdom Renal Imaging Network Kidney Analysis Toolbox (UKAT) [35].

- *Quantifying T*_*2*_ *‐mapping Accuracy*: Mean T_2_ in each ISMRM/NIST T_2_‐array sphere was compared to the reference value.
- *Evaluating Diffusion Effects*: Mean T_2_ in the “capillary bed” of the QASPER phantom was calculated at each flow rate to evaluate sensitivity to diffusion.
- *Evaluating In Vivo Renal Cortex and Medulla T*_*2*_: Cortex and medulla regions of interest (ROIs) were manually defined from T_1_‐weighted scans by the researcher (AD) using an interactive graphical interface in MRIcron (v12.12.2012), and ROI accuracy was confirmed by another researcher (SF). Mean and standard deviation of cortex and medulla T_2_ were calculated for each participant, after exclusion of voxels with *R* ^2^ < 0.8.

### 2.6 Statistical Analysis

Statistical analysis was performed using Python packages Pingouin (v0.5.3) and Statsmodels (v0.13.2); a *p*‐value of < 0.05 was considered statistically significant. T_2_‐mapping data were tested for normality using a Shapiro–Wilk test.

For each T_2_‐mapping sequence, accuracy was quantified from the ISMRM/NIST T_2_ array. Mean T_2_ values for each sphere were compared to the reference values using the Mean Absolute Scaled Error (MASE) $=\frac{\frac{1}{N}\sum_{n}\left|T_{2\;n}^{\text{ground truth}}-T_{2\;n}^{\text{estimate}}\right|}{\frac{1}{N}\sum_{n}\left|T_{2\;n}^{\text{ground truth}}-\bar{T_{2}^{\text{ground truth}}}\right|}$ where N is the number of spheres, $T_{2\;n}^{\text{ground truth}}$ is the reference value of each sphere, $T_{2\;n}^{\text{estimate}}$ is the mean estimated T_2_ of a sphere and $\bar{T_{2}^{\text{ground truth}}}$ is the mean reference T_2_ of all spheres [36]. MASE was chosen over Mean Absolute Percentage Error (MAPE) as MASE penalizes both over and underestimation equally and weights errors in spheres with a short T_2_ equally to those with a long T_2_ [37]. MASE was calculated over both the full T_2_ array range (5–650 ms) and the kidney T_2_ range (40–200 ms) [12]. For the QASPER phantom, the significance of changes in T_2_ with diffusion was tested using a Wilcoxon signed‐rank test.

In vivo, parametric tests were used to compare T_2_ values measured across sequences, and the Coefficient of Variation (CoV) between participants was calculated. Bland–Altman plots assess agreement between T_2_‐mapping methods. Pearson's correlation coefficient (*R*) assessed the influence of average ΔB_0_ and B_1_^+^ on measured T_2_. Voxelwise sensitivity between ΔB_0_/B_1_ and T_2_ in the cortex and medulla was assessed using a random intercept linear mixed effects model. The intercept for each participant/tissue combination was modeled as a random effect to allow for physiological variations of T_2_ in each participant/tissue type. Given the symmetrical nature of B_1_^+^ related errors in T_2_‐mapping, that is, voxels with B_1_^+^ of 80% and 120% of nominal flip angle result in the same T_2_, B_1_^+^ values were reflected about 100% ($B_{1\;\text{reflected}}=100-\left|100-B_{1\;\text{measured}}\right|$) before performing statistical tests.

Bonferroni correction was used when comparing the four T_2_‐mapping sequences to minimize type I errors.

## 3 Results

### 3.1 Assessing Signal Intensity

**FIGURE 3:**
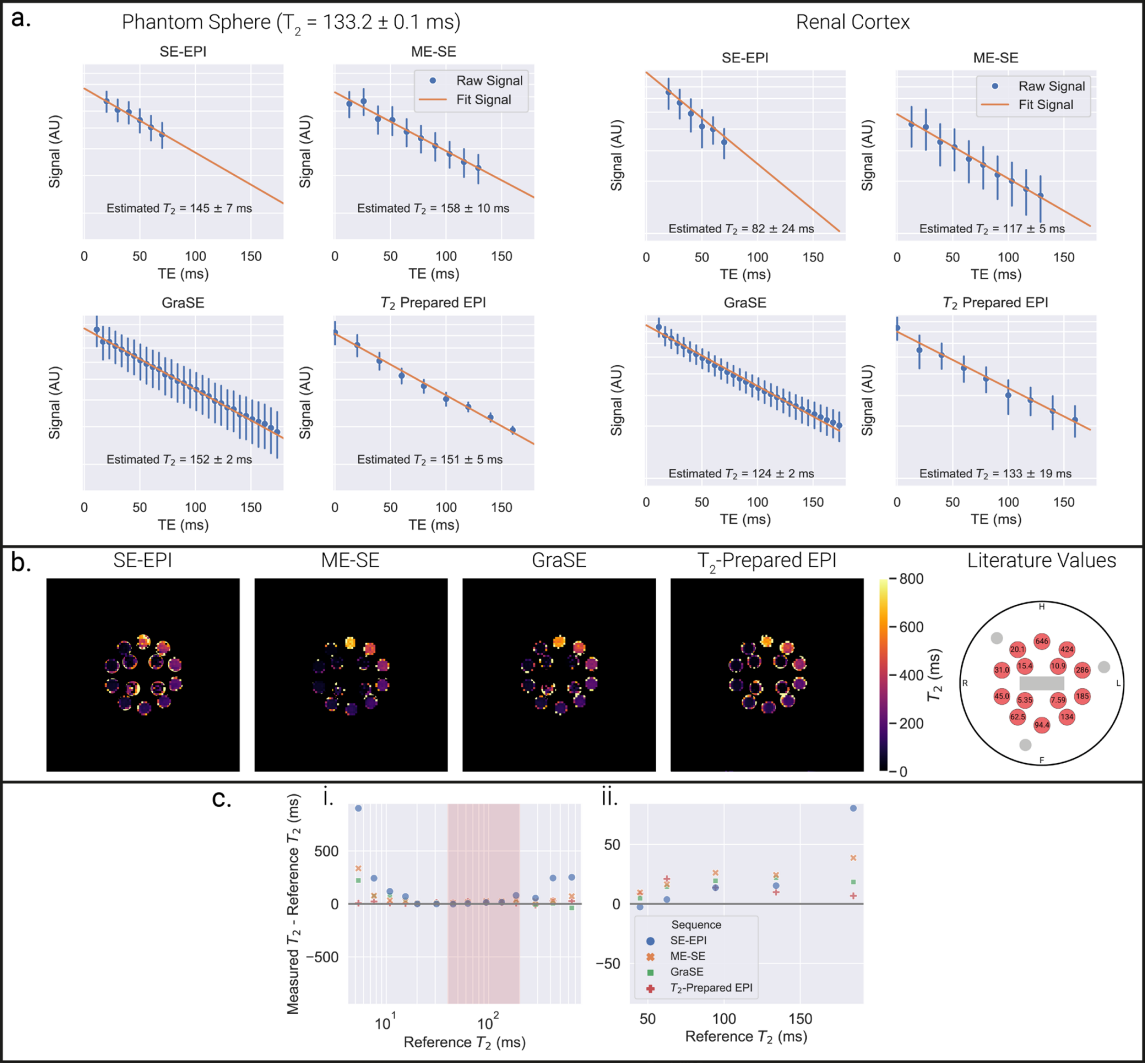
(a) Signal intensity versus echo time for the ISMRM/NIST T_2_ sphere with reference value of 133.2 ± 0.1 ms and renal cortex of Participant 7 (example maps shown in Figure 6). Signals are presented for the SE‐EPI, ME‐SE, GraSE (which has one start‐up echo and a larger number of echoes) and T_2_‐prepared EPI sequences. Stimulated echo effects are seen in both the ME‐SE and GraSE sequences—the ME‐SE sequence has a higher signal intensity for the second echo than the first, the GraSE sequence has one “start‐up echo” meaning data acquisition starts at the higher second echo signal. Data are presented on a logarithmic *y*‐axis to highlight any deviation from the mono‐exponential decay of the fit signal. Error bars represent the standard deviation over the region of interest. (b) T_2_ maps of the ISMRM/NIST phantom T_2_ array for each T_2_ mapping sequence and the reference values. (c) Average T_2_ measured within each sphere compared to the reference T_2_ values shown for each T_2_ mapping sequence for the (i) full range of T_2_ spheres on a logarithmic *x*‐axis with physiological kidney T_2_ range shaded in red, and (ii) spheres with reference T_2_ values in the physiological kidney T_2_ range shown on a linear *x*‐axis.

Example signal intensity versus TE plots are shown in Figure 3a for an ISMRM/NIST T_2_ sphere (133.2 ± 0.1 ms) and renal cortex. The signal intensity for all echoes was substantially above the noise floor; the data for the ME‐SE and GraSE sequences show stimulated echo effects.

### 3.2 Quantifying T_2_ Accuracy in ISMRM/NIST Phantom

Example T_2_ maps of the ISMRM/NIST T_2_ array for each T_2_‐mapping sequence are shown in Figure 3b. Figure 3c compares the measured to the reference sphere T_2_. All T_2_‐mapping sequences had limited accuracy for the very short (5.35–15.4 ms, thus non‐physiological) T_2_ spheres, with the T_2_‐prepared EPI method being most accurate. The SE‐EPI T_2_‐mapping sequence overestimated the T_2_ of both the short (5–40 ms) and long (185–646 ms) T_2_ spheres but matched values over the physiological T_2_ kidney range (40–200 ms). Supporting Information S1 shows the influence of the choice of echo times on SE‐EPI T_2_ measurements; results show that for T_2_ spheres within the physiological kidney range, the choice of echo times has limited effect on the measured T_2_. The SE‐EPI, ME‐SE, GraSE, and T_2_‐prepared EPI sequences resulted in a MASE of 1.02, 0.37, 0.28, and 0.09, respectively, over the full range of T_2_ spheres. Note MASE values are scaled by the mean reference T_2_ of each range, thus values can be compared within each range, but not across ranges. For T_2_ spheres in the kidney T_2_ range, the T_2_‐prepared EPI sequence was most accurate, while ME‐SE and GraSE sequences overestimated T_2_, as shown in Figure 3a. This is reflected in the MASE of 0.52, 0.52, 0.36, and 0.27 for SE‐EPI, ME‐SE, GraSE, and T_2_‐prepared EPI, respectively, over the kidney T_2_ range.

### 3.3 Influence of B_0_ and B_1_^+^ on ISMRM/NIST Phantom T_2_ Array

**FIGURE 4:**
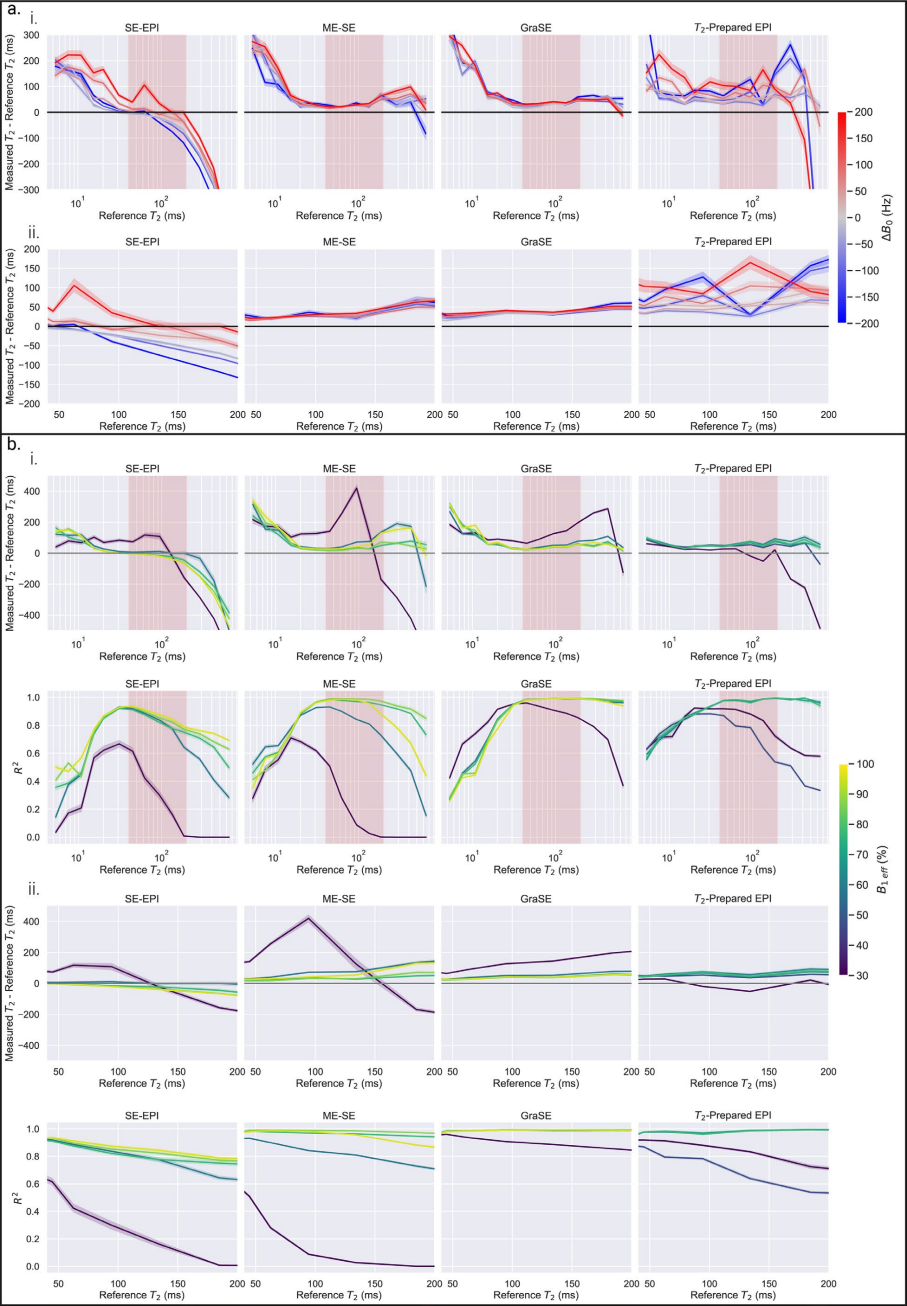
(a) Measured compared to ISMRM/NIST reference T_2_ for different B_0_ offsets over the (i) full range of T_2_ spheres with the physiological T_2_ kidney range shaded red and (ii) spheres with reference T_2_ values in the physiological kidney range. (b) Measured compared to reference T_2_ and associated *R* ^2^ values for a range of effective B_1_ shown over the (i) full range of T_2_ spheres and (ii) spheres with the physiological range. Error bars are 95% confidence intervals calculated from the variance in measured T_2_ within each sphere.

Figure 4a shows the influence of the B_0_ field on the measured T_2_ of the T_2_ array; SE‐EPI and T_2_‐prepared EPI sequences displayed the largest variation with ΔB_0_. Figure 4b shows the effect of controlled modulation of B_1_^+^ on the measured T_2_ of the T_2_ array. Using B_1eff_ < 50%, all sequences were very inaccurate (MASE: 1.47, 2.25, 1.12, and 0.63 for SE‐EPI, ME‐SE, GraSE, and T_2_‐prepared EPI respectively over the full T_2_ array range) (Figure 4b(i)). For a typical B_1eff_ measured in the kidneys (90%–105% [38]), of the four T_2_‐mapping sequences, the T_2_ measured using ME‐SE varied most with B_1eff_ over the kidney T_2_ range (Figure 4b(ii)), with the lowest *R* ^2^ measured in the kidneys with SE‐EPI and T_2_‐prepared EPI. Supporting Information S2 shows T_2_ array signal intensity versus echo time plots as a function of B_1eff_.

### 3.4 Sensitivity of T_2_‐Mapping Sequences to Diffusion in QASPER Phantom

**FIGURE 5:**
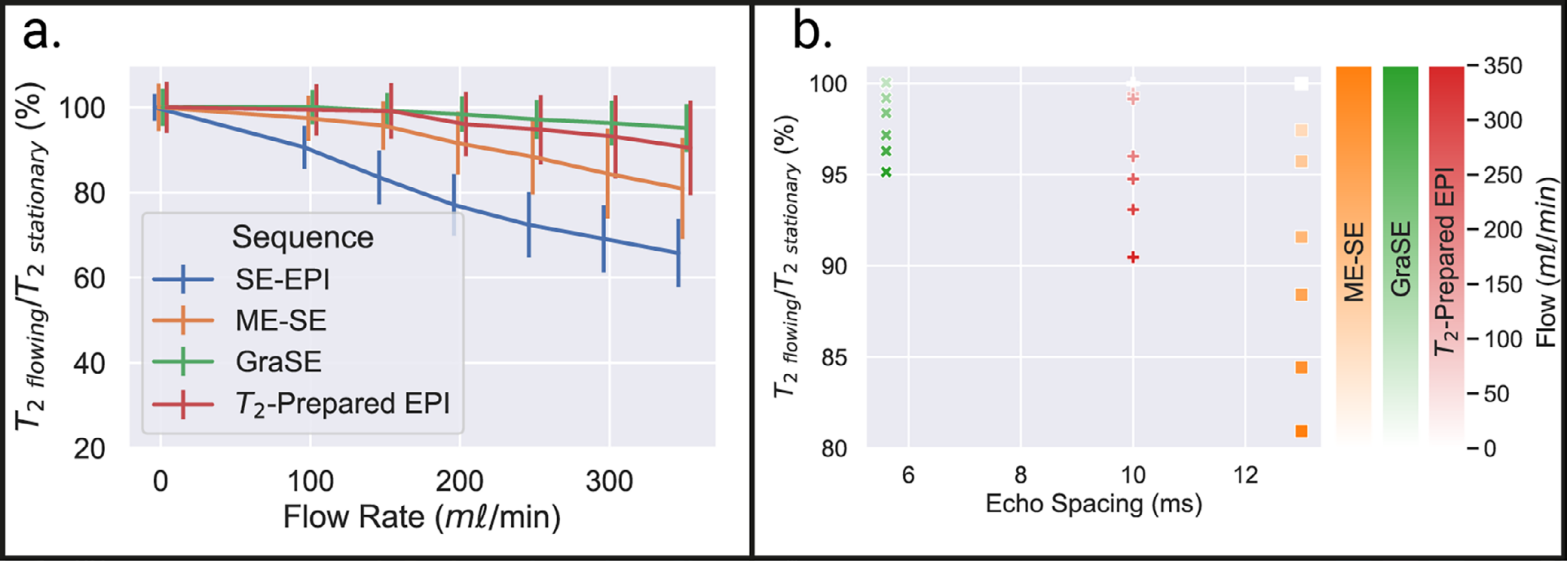
(a) QASPER phantom data showing the effect of increasing perfusate flow rate through the simulated capillary bed on T_2_ as a percentage of T_2_ measured with the pump switched off for each T_2_ mapping sequence. Error bars show the standard deviation of values within the “capillary bed.” Points are staggered on the *x*‐axis to aid the visibility of error bars. (b) Reduction in measured T_2_ on increasing flow rate shown for each echo spacing of the multi‐echo sequence.

The percentage change in T_2_ in the “capillary bed” with diffusion compared to with the pump switched off is shown in Figure 5a. SE‐EPI was most sensitive to diffusion with the largest T_2_ reduction as flow rate increased. For multi‐echo sequences, measured T_2_ depended on echo spacing, with the GraSE sequence having the shortest echo spacing and minimal sensitivity to diffusion, Figure 5b. For all flow rates and sequences, a significant difference in T_2_ was observed between the pump on and off, except for the GraSE sequence with 100 mL/min (*p* = 0.24).

### 3.5 In Vivo T_2_ Measures

**FIGURE 6:**
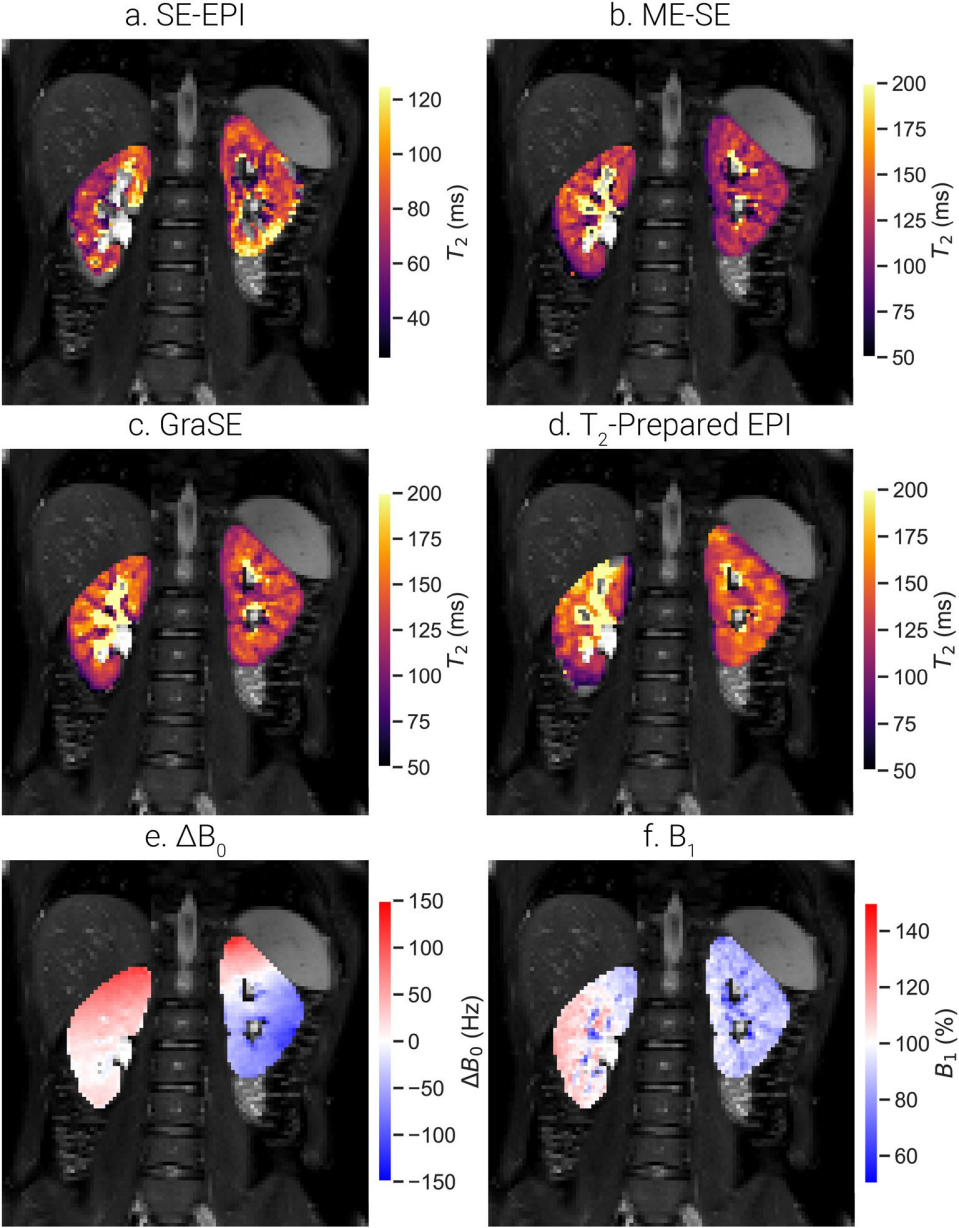
Example in vivo T_2_ maps using (a) SE‐EPI, (b) ME‐SE, (c) GraSE and (d) T_2_‐prepared EPI sequences, (e) ΔB_0_ map and (f) B_1_ map (data shown for Participant 7). Note the scale of the color bar used for the SE‐EPI T_2_ map.

Example in vivo T_2_ maps for each sequence and ΔB_0_ and B_1_ maps are shown in Figure 6. GraSE and T_2_‐prepared EPI T_2_ maps showed the largest contrast between cortex and medulla, while T_2_‐prepared EPI and SE‐EPI maps had visible field inhomogeneities and blurring.

**FIGURE 7:**
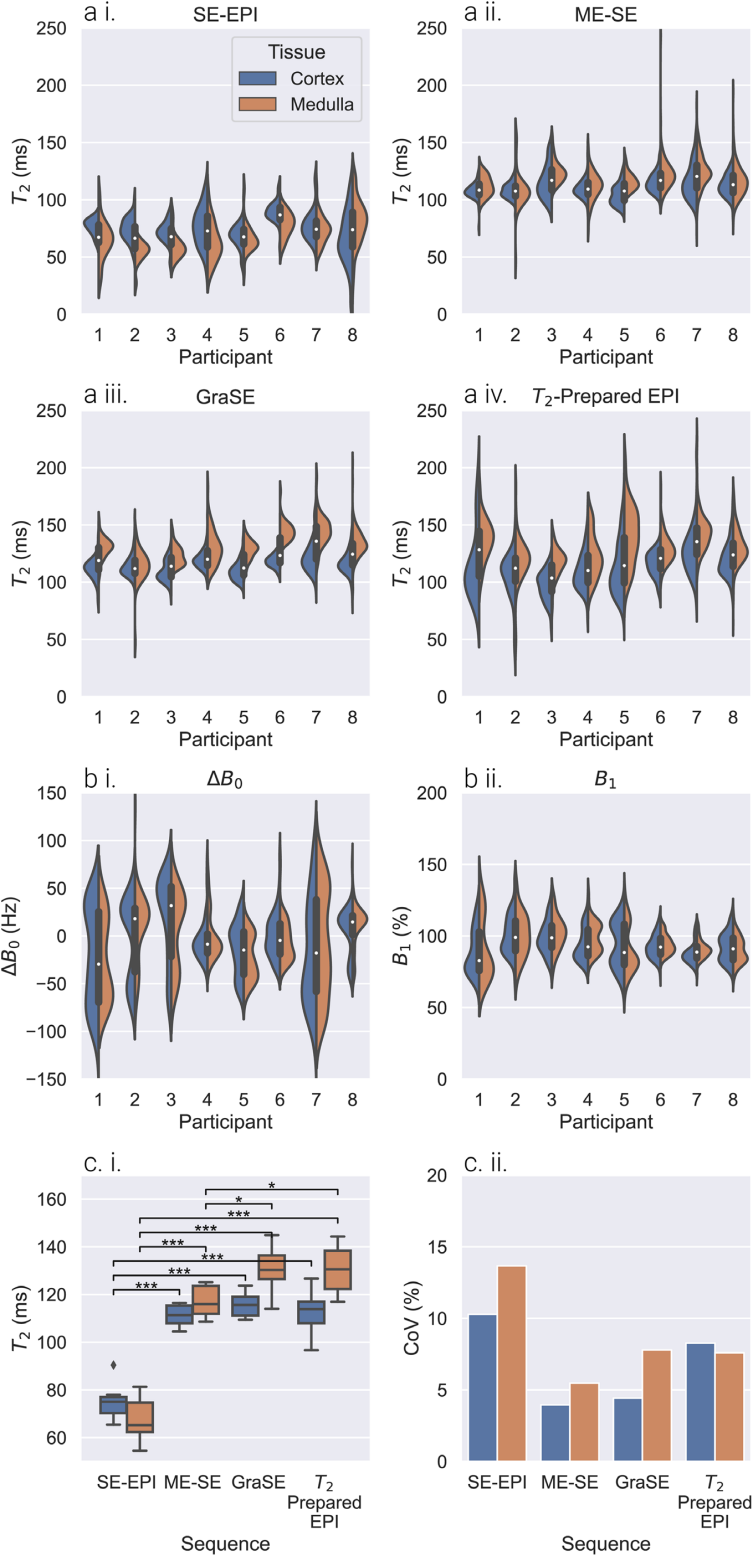
(a) Violin plots of T_2_ measured in cortex (blue) and medulla (orange) of each participant for (i) SE‐EPI, (ii) ME‐SE, (iii) GraSE, and (iv) T_2_‐prepared EPI. (b) (i) ΔB_0_ and (ii) B_1_ in each participant. (c) Result for each T_2_ mapping method showing (i) box plot of mean T_2_ and (ii) bar chart of CoV in T_2_ across all participants.

Figure 7 shows violin plots of the distribution of T_2_ values within cortex and medulla for each participant, together with mean and CoV across participants. In line with the QASPER phantom data, the SE‐EPI sequence measured a significantly lower T_2_ (75 ± 8 and 65 ± 9 ms for cortex and medulla) compared to multi‐echo sequences (ME‐SE 111 ± 4 and 116 ± 6 ms, GraSE 116 ± 5 and 130 ± 10 ms, and T_2_‐prepared EPI 114 ± 9 and 131 ± 10 ms for cortex and medulla), particularly in medulla ROIs for which the GraSE and T_2_‐prepared EPI sequences measured a significantly higher T_2_ than the ME‐SE sequence. The lowest CoV across participants was seen for ME‐SE, and was greatest for SE‐EPI due to both larger variance across participants and T_2_ shortening due to diffusion.

**FIGURE 8:**
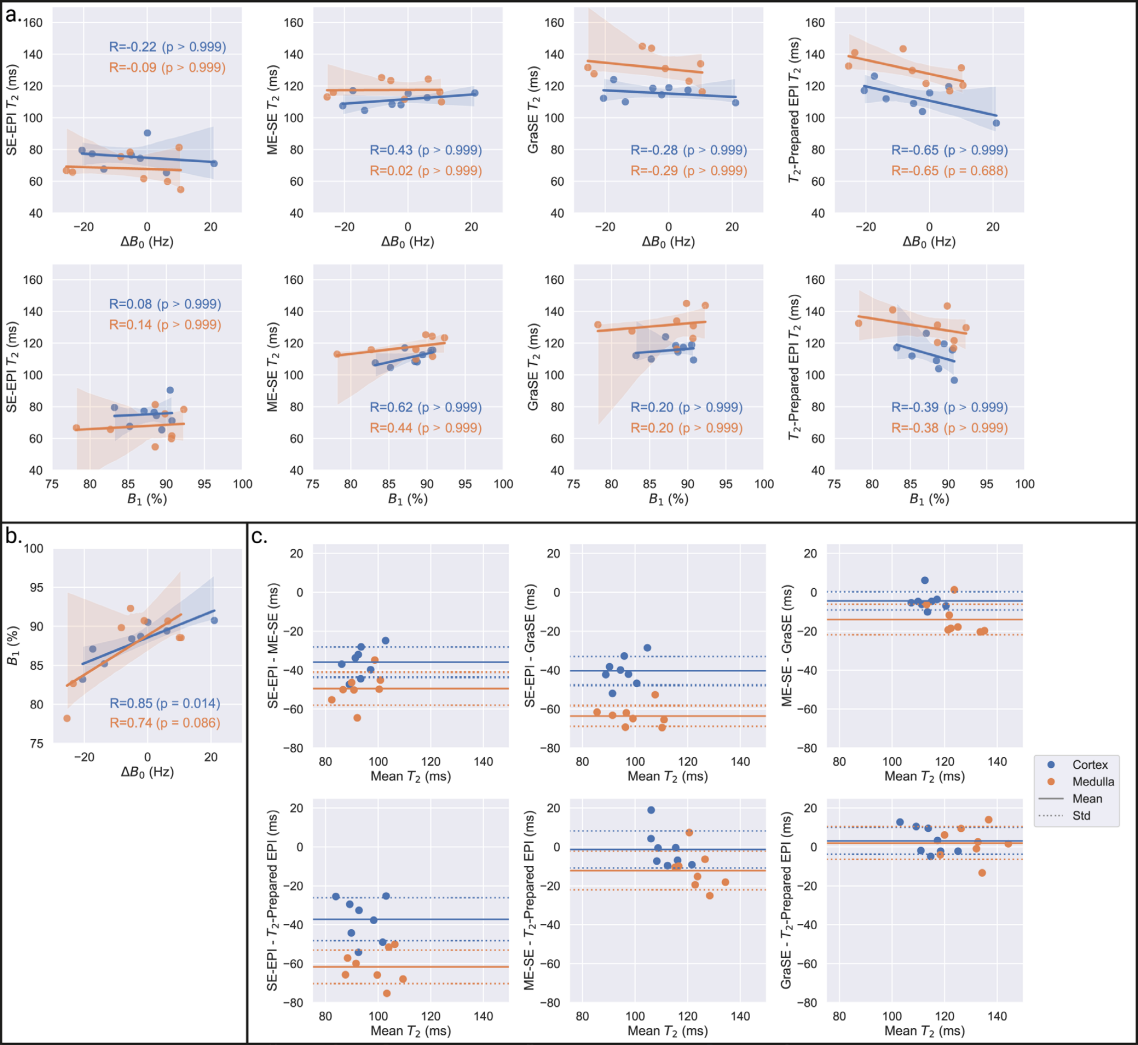
(a) For each T_2_ mapping sequence the correlation between measured T_2_ and ΔB_0_, and measured T_2_ and B_1_ for cortex and medulla. (b) Correlation between ΔB_0_ and B_1_. For each the Pearson correlation coefficient (*R*) is provided for renal cortex (blue), medulla (orange). (c) Bland–Altman plots showing the agreement in average cortex and medulla T_2_ between each T_2_ mapping method.

Figure 8a shows that the mean T_2_ measured in cortex and medulla (averaged across left and right kidneys) had no significant correlation with ΔB_0_ and B_1_ (*p* = 0.688 to > 0.999), there was a significant correlation between mean ΔB_0_ and B_1_ in cortex but not the medulla (*p* = 0.086), Figure 8b. Figure 8c shows Bland–Altman plots of the bias between T_2_ measured with each sequence.

**FIGURE 9:**
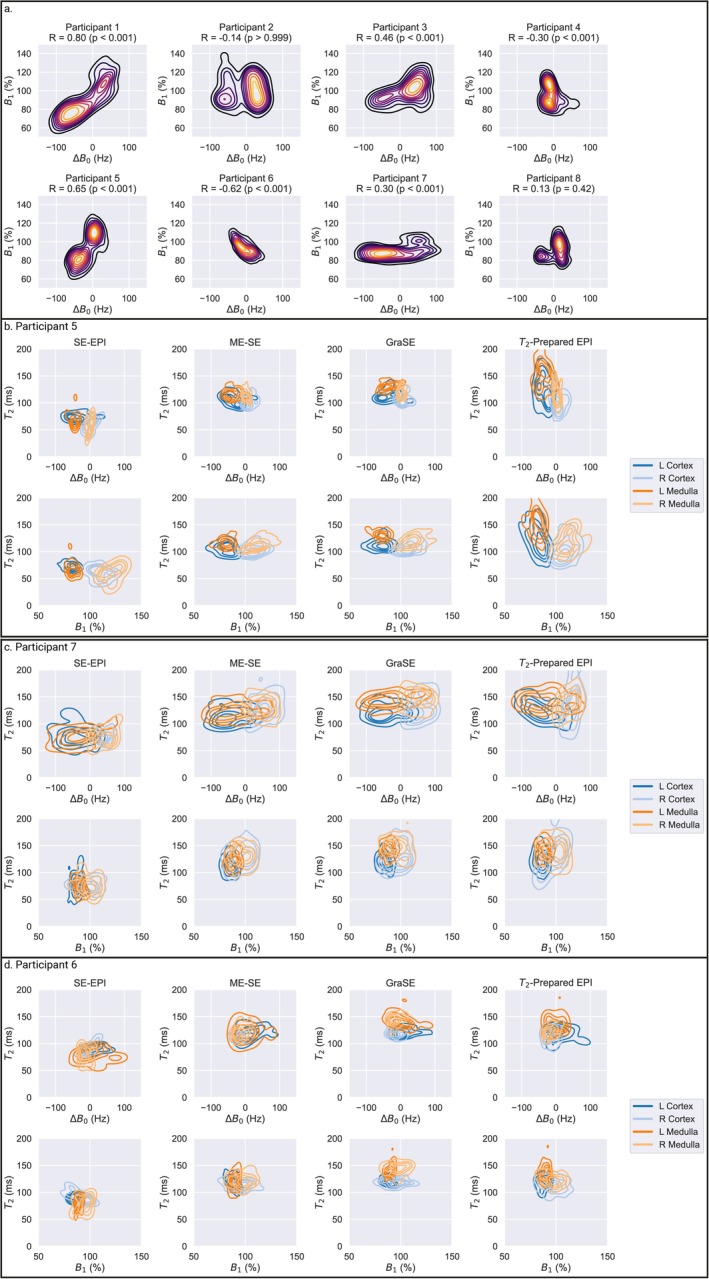
(a) Contour plots showing the correlation between ΔB_0_ and B_1_^+^ for all participants, brighter contours (yellow) represent a large number of voxels and darker contours (black) a smaller number of voxels. (b–d) Correlation between measured T_2_ and ΔB_0_ and B_1_ for each T_2_ mapping method with the left/right cortex/medulla shown as different colored contours. (b) Example participant with a large range in B_1_^+^, (c) example participant with a large range in ΔB_0_, and (d) example participant with a small range in ΔB_0_ and B_1_^+^.

Figure 9a demonstrates a considerable variation in ΔB_0_ and B_1_ on a voxel‐wise basis within the kidney across participants. Figure 9b–d shows those participants which illustrate relationships between the measured T_2_ and underlying B_0_ and B_1_ fields, with the T_2_‐prepared EPI sequence being highly sensitive to B_0_ and B_1_^+^ field inhomogeneity. In Figure 9b, Participant 5 has a low range in ΔB_0_ within the kidney but a large distribution of the B_1_^+^ field resulting in a V‐shape B_1_^+^ effect on the T_2_ for T_2_‐prepared EPI, and to a lesser extent ME‐SE and GraSE, as T_2_ is overestimated when RF pulses are away from their nominal (100%) value. Participant 7 shown in Figure 9c had a lower distribution in B_1_^+^ field within the kidney but large ΔB_0_ fluctuations, leading to broadening of the distribution of T_2_ values for SE‐EPI and T_2_‐prepared EPI sequences. While Figure 9d shows Participant 6 with a narrow distribution in both ΔB_0_ and B_1_^+^ field across the kidneys, leading to a tighter range in T_2_ values compared to Participants 5 and 7. The linear mixed effects model of ΔB_0_ and B_1_ field showed that the ME‐SE and T_2_‐prepared EPI sequences had a significant but small dependence on ΔB_0_ (0.030 ± 0.008 and −0.07 ± 0.01 ms/Hz, respectively). All sequences had a significant dependence on B_1_, with a larger effect on T_2_ measured using the SE‐EPI and T_2_‐prepared EPI sequences (−0.52 ± 0.08 and −0.57 ± 0.06 ms/%, respectively) than ME‐SE and GraSE sequences (−0.10 ± 0.04 and −0.11 ± 0.04 ms/%, respectively).

## 4 Discussion

This study assessed four renal T_2_‐mapping sequences for the influence of imaging parameters on measured T_2_ accuracy, sensitivity to diffusion, and B_0_/B_1_^+^ fields.

### 4.1 Accuracy of Measured T_2_ in the ISMRM/NIST Phantom

Over the kidney range of the T_2_ array (40–200 ms), all sequences performed well, while across the full range of the T_2_ array (5–646 ms) the T_2_‐prepared EPI sequence was most accurate since this had an effective echo time of 0 ms to sample the decay of the short T_2_ spheres. SE‐EPI, ME‐SE, and GraSE measures all had a large bias in measured T_2_ for the shortest (< 20 ms) T_2_ spheres, with SE‐EPI having the most pronounced bias and overestimating the T_2_ of the long spheres (185–646 ms) due to the relatively late first TE and limited number of echo times and thus TE range. ME‐SE and GraSE (to a lesser extent due to the inclusion of one start‐up echo) sequences overestimated T_2_ due to fitting a pure exponential curve to the experimental multi‐echo echo‐modulation curve [39].

### 4.2 Sensitivity of T_2_ ‐Mapping Sequences to Diffusion

In the QASPER phantom, SE‐EPI T_2_‐mapping was highly sensitive to diffusion, resulting in a large reduction in measured T_2_ for higher flow rates. As expected, the other sequences (GraSE, T_2_‐prepared EPI, and ME‐SE) had less diffusion dependence [40], with the sequence with the shortest echo spacing (GraSE < T_2_‐prepared < ME‐SE) having the least diffusion sensitivity. This effect of echo spacing can be interpreted through R1ρ or CPMG dispersion frameworks, with shorter echo spacing increasing the effective spin‐lock strength, resulting in more efficient suppression of T_2_ components from slow water motion [41].

In vivo, kidney T_2_‐mapping was influenced by diffusion, leading to statistically significant shorter cortex and medulla measured T_2_ values for SE‐EPI and the largest CoV between participants compared to multi‐echo sequences. For renal cortex, there was no significant difference in measured T_2_ using the ME‐SE, GraSE, and T_2_‐prepared EPI sequences. In contrast, in the medulla, measured T_2_ was ordered with significantly higher values and lower between‐participant CoV for sequences with shorter echo spacing (and therefore reduced diffusion effects in line with the QASPER phantom results). Although the cortex is more highly perfused than the medulla [1], the sensitivity of medullary T_2_ to echo spacing and diffusion effects can be attributed to the high renal tubular density in medullary pyramids with fluid flow through renal tubules and blood vessels traversing to the medulla [1].

Our measured multi‐echo kidney T_2_ values of 111–116 ms for cortex and 116–131 ms for medulla are in line with literature (Table S1) values measured for T_2_‐prepared sequences of 121 ± 5 and 138 ± 7 ms for cortex and medulla [21], 113 ± 8 ms for cortex [42], and 111 ± 5 and 116 ± 8 ms for cortex and medulla [24]. While de Bazelaire et al. [16] measured shortened T_2_ values of 76 ± 7 and 81 ± 8 ms for cortex and medulla using a SE‐SSFP sequence, similar to our reported SE‐EPI T_2_ measures. Due to the diffusion sensitivity of SE‐EPI T_2_‐mapping, it is not recommended for the study of kidney pathophysiology. However, in future studies, collection of ME‐SE T_2_‐mapping data at different echo spacings or alongside diffusion weighted imaging or T_1_ρ [43] could improve interpretation by isolating diffusion/flow‐related T_2_ components from edema/inflammation T_2_ components.

### 4.3 Sensitivity of T_2_ ‐Mapping Sequences to B_0_ and B_1_ ^+^ Field

The T_2_‐mapping sequences had different sensitivity to B_0_ and B_1_^+^ inhomogeneity. In the ISMRM/NIST phantom, the SE‐EPI and T_2_‐prepared EPI sequences were most sensitive to B_0_, with off‐resonance dependencies in both the T_2_‐preparation echo train and EPI readout. For B_1_^+^ fields adjusted to < 50% of nominal flip angle, all sequences were inaccurate. For SE‐EPI T_2_‐mapping, any B_1_^+^ inhomogeneity alters each refocusing and excitation angle, and theoretically, the measured T_2_ should not change, though variance will increase. However, our SE‐EPI T_2_‐mapping data showed an increase in measured T_2_ in the shorter spheres as B_1_^+^ reduced because the vendor‐specific sequence altered the profile of excitation and refocusing pulse used for the first TE compared to later echoes.

Although modulating the B_0_/B_1_^+^ fields in a spherical phantom enables a highly controlled dependence on T_2_‐mapping, in vivo B_0_/B_1_^+^ fields are more spatially variable. Patient size, body shape, and geometry of abdominal organs and lungs result in substantial local heterogeneity in B_0_/B_1_^+^, particularly at 3 T compared to 1.5 T [44]. When averaging voxels within a ROI to study the effects of B_0_/B_1_^+^ fields, this can mask underlying variability as compared to studying voxel‐wise measures. In vivo, voxel‐wise, the T_2_‐prepared EPI sequence was highly sensitive to B_0_ and B_1_^+^ field inhomogeneity, resulting in the second largest CoV across participants. This was despite this sequence using non‐selective composite pulses with MLEV phase cycling for relative insensitivity to B_1_ inhomogeneity [45]. Recent studies have demonstrated that while composite pulses with MLEV phase cycling mitigate B_1_^+^ effects, errors in the quantification of T_2_ can remain in the presence of large inhomogeneities, particularly at higher fields [46, 47], and that adiabatic BIR‐4 RF pulses provide more accurate B_1_‐insensitive T_2_ values [48]. For ME‐SE sequences, signal contamination due to imperfect 180° refocusing pulses leads to stimulated and indirect echo pathways that prolong the signal decay, leading to T_2_ overestimation [39]. However, these effects were small in the in vivo datasets in this study, likely due to B_0_ and B_1_ shimming over the kidney and the low BMI of healthy participants.

## 5 Limitations

Future studies should systematically test the effect of echo‐spacing, perform EPG modeling for ME‐SE sequences to compensate for stimulated echoes [23], and use of adiabatic pulses for T_2_‐preparation to reduce sensitivity to B_1_^+^ inhomogeneities [49]—although this may introduce a potential bias [50]. In this study, in vivo comparisons were performed on a small healthy participant population with normal BMI. The shorter echo‐spacing of GraSE allows a higher number of echoes to be sampled than for ME‐SE, but this results in a higher specific absorption rate (SAR). When scanning larger BMI participants, the high SAR of the GraSE sequence can result in the acquisition being divided into more packages/shots, increasing acquisition time.

## 6 Conclusion

A vendor‐provided multi‐echo sequence with a short echo spacing to reduce sensitivity to flow/diffusion and a range of echo times up to the expected kidney T_2_ values for accuracy is recommended. Collecting data at different echo spacings may provide a method by which to isolate edema/inflammation from diffusion‐related changes to offer specificity for clinical applications.

## Supporting information


**Data S1:** jmri70127‐sup‐0001‐supinfo.docx.
