# Supplementary material for: Assessing the Impact of Imaging Parameters on MRI Measurement of Kidney T2
Source: J Magn Reson Imaging. 2025 Sep 25;63(2):508–22. doi: 10.1002/jmri.70127 (PMC12810998; doi:10.1002/jmri.70127)
Supplement: Supplementary file 1 — Data S1: jmri70127‐sup‐0001‐supinfo.docx. [file JMRI-63-508-s001.docx]

| **Author** | **Year** | **Group** | **Sub-Group** | **Sample (n)** | **T_2_ mapping sequence** | **CortexT_2_ (ms)** | **Medulla T_2_ (ms)** |
| --- | --- | --- | --- | --- | --- | --- | --- |
| **1.5T** | | | | | | | |
| de Bazelaire *et al.*^1^ | 2004 | Healthy | - | 4 | SE-SSFP | 87 ± 4 | 85 ± 11 |
| Zhang *et al*.^2^ | 2011 | Healthy | Visit 1 | 4 | ME-FSE | 112 ± 8 | 137 ± 13 |
|  |  |  | Visit 2 | 4 |  | 112 ± 8 | 143 ± 14 |
| Mathys *et al.*^3^ | 2011 | Healthy |  | 6 | ME-SE | 125 ± 7 | - |
|  |  | Renal allograft | GFR > 40 | 6 |  | 147 ± 13 |  |
|  |  | Renal allograft | GFR < 40 | 9 |  | 150 ± 20 |  |
| Siedek *et al*.^4^ | 2020 | Healthy | - | 10 | GraSE | 121 ± 11 | - |
|  |  | ADPKD | - | 139 |  | 275 ± 63 |  |
| Dillman *et al.*^5^ | 2022 | Healthy | - | 20 | ME-FSE | 112.7 (108.6-117.2)* | 127.7 (124.1-133.6)* |
|  |  | CKD | - | 12 |  | 116.1 (109.1-124.7)* | 128.7 (123.5-131.1)* |
| Keerthivasan *et al*.^6^ | 2022 | Liver disease | - | 12 | Constant Flip Angle Radial FSE |  | 114 ± 8.6 |
|  |  |  |  |  | Variable Flip Angle Radial FSE |  | 113 ± 8.7 |
| **3T** | | | | | | | |
| de Bazelaire *et al.*^1^ | 2004 | Healthy | - | 6 | SE-SSFP | 76 ± 7 | 81 ± 8 |
| Li *et al*.^7^ | 2015 | Healthy | - | 5 | CPMG T_2_ Prep | 121 ± 5 | 138 ± 7 |
| Franke *et al*.^8^ | 2017 | Healthy | - | 3 | GraSE | 132 ± 6 | - |
|  |  | ADPKD | TKV < 300 ml | 3 |  | 417 ± 65 |  |
|  |  |  | TKV 300–400 ml | 3 |  | 592 ± 231 |  |
|  |  |  | TKV > 400 ml | 3 |  | 669 ± 170 |  |
| Adams et al ^9^ | 2019 | Renal Cell Carcinoma | - | 16 | CPMG T_2_ Prep | 85 ± 16 | 92 ± 16 |
| Adams *et al.*^10^ | 2020 | Healthy |  | 16 | T_2_ Prep | 78 ± 4 | 59 ± 2 |
|  |  | Renal allograft | Early Transplant | 22 |  | 83 ± 6 | 67 ± 5 |
|  |  | Renal allograft | Late Transplant | 24 |  | 82 ± 8 | 62 ± 3 |
| de Boer *et al*.^11^ | 2020 | Healthy | Visit 1 | 34 | MLEV T_2_ Prep | 113 ± 8 | - |
|  |  |  | Visit 2 |  |  | 114 ± 8 |  |
| Li *et al.*^12^ | 2024 | Healthy | GE | 8 | ME-SE | 109 ± 5 | 114 ± 7 |
|  |  |  | Philips | 8 |  | 111 ± 5 | 116 ± 8 |
|  |  |  | Siemens | 8 |  | 125 ± 8 | 129 ± 8 |
| Horvat-Menih *et al.*^13^ | 2024 | Renal Cell Carcinoma | - | 24 | High-res TEMPURA | 110 (88 – 124)^†^ | - |
|  |  |  |  |  | Breath Hold TEMPURA | 108 (89 – 125) ^†^ |  |
| Henry *et al.*^14^ | 2024 | Healthy |  | 125 | GraSE | 98 ± 7 | - |
|  |  | Renal Masses | Carcinoma | 39 |  | 105 ± 8 |  |
|  |  |  | Angiomyolipomas | 25 |  | 117 ± 6 |  |
|  |  |  | Oncocytomas | 11 |  | 85 ± 4 |  |

***Table S1:*** *Updated version of Table 2 from Wolf et al.*^15^ *showing studies measuring renal T_2_ in human participants and their acquisition sequences (excluding MR fingerprinting methods). T_2_ values quoted as mean ± standard deviation aside from * which is quoted as mean and interquartile range and ^†^ which is quoted as median and range. Studies were identified using the PubMed query: (‘‘kidney’’[MeSH Terms] OR ‘‘kidney’’[All Fields] OR ‘‘kidneys’’[All Fields]) AND (‘‘cortex’’[All Fields] OR ‘‘medulla’’[All Fields] OR ‘‘parenchyma’’[All Fields]) AND (‘‘T2 mapping’’[All Fields] OR ‘‘T2 map’’ [All Fields] OR (‘‘T2’’[All Fields] AND (‘‘mapping’’[All Fields] OR ‘‘quantification’’[All Fields]))) AND (‘‘magnetic resonance imaging’’[MeSH Terms] OR (‘‘magnetic’’[All Fields] AND ‘‘resonance’’[All Fields] AND ‘‘imaging’’[All Fields]) OR ‘‘magnetic resonance imaging’’[All Fields] OR ‘‘mri’’[All Fields]) AND (‘‘human’’[All Fields] OR ‘‘humans’’[MeSH Terms]) AND (2000/01/01:2025/12/31[Date - Publication] AND ‘‘English’’[Language])*

**References**

1. de Bazelaire CMJ, Duhamel GD, Rofsky NM, Alsop DC. MR Imaging Relaxation Times of Abdominal and Pelvic Tissues Measured in Vivo at 3.0 T: Preliminary Results. *Radiology*. 2004;230(3):652-659. doi:10.1148/radiol.2303021331

2. Zhang JL, Storey P, Rusinek H, et al. Reproducibility of R2* and R2 Measurements in Human Kidneys. In: *Proc. Intl. Soc. Mag. Reson. Med. 19*. Vol 19. ; 2011:2954. https://cds.ismrm.org/protected/11MProceedings/files/2954.pdf.

3. Mathys C, Blondin D, Wittsack HJ, et al. T2’ Imaging of Native Kidneys and Renal Allografts – a Feasibility Study. *RöFo - Fortschritte Auf Dem Geb Röntgenstrahlen Bildgeb Verfahr*. 2011;183(2):112-119. doi:10.1055/s-0029-1245597

4. Siedek F, Grundmann F, Weiss K, et al. Magnetic Resonance Kidney Parenchyma-T2 as a Novel Imaging Biomarker for Autosomal Dominant Polycystic Kidney Disease. *Invest Radiol*. 2020;55(4):217-225. doi:10.1097/RLI.0000000000000633

5. Dillman JR, Benoit SW, Gandhi DB, et al. Multiparametric quantitative renal MRI in children and young adults: comparison between healthy individuals and patients with chronic kidney disease. *Abdom Radiol*. 2022;47(5):1840-1852. doi:10.1007/s00261-022-03456-x

6. Keerthivasan MB, Galons JP, Johnson K, et al. Abdominal T2-Weighted Imaging and T2 Mapping Using a Variable Flip Angle Radial Turbo Spin-Echo Technique. *J Magn Reson Imaging*. 2022;55(1):289-300. doi:10.1002/jmri.27825

7. Li X, Bolan PJ, Ugurbil K, Metzger GJ. Measuring renal tissue relaxation times at 7 T. *NMR Biomed*. 2015;28(1):63-69. doi:10.1002/nbm.3195

8. Franke M, Baeßler B, Vechtel J, et al. Magnetic resonance T2 mapping and diffusion-weighted imaging for early detection of cystogenesis and response to therapy in a mouse model of polycystic kidney disease. *Kidney Int*. 2017;92(6):1544-1554. doi:10.1016/j.kint.2017.05.024

9. Adams LC, Bressem KK, Jurmeister P, et al. Use of quantitative T2 mapping for the assessment of renal cell carcinomas: first results. *Cancer Imaging*. 2019;19(1):35. doi:10.1186/s40644-019-0222-8

10. Adams LC, Bressem KK, Scheibl S, et al. Multiparametric Assessment of Changes in Renal Tissue after Kidney Transplantation with Quantitative MR Relaxometry and Diffusion-Tensor Imaging at 3 T. *J Clin Med*. 2020;9(5):1551. doi:10.3390/jcm9051551

11. de Boer A, Harteveld AA, Stemkens B, et al. Multiparametric Renal MRI: An Intrasubject Test–Retest Repeatability Study. *J Magn Reson Imaging*. 2020;53(3):859-873. doi:10.1002/jmri.27167

12. Li H, Daniel AJ, Buchanan CE, et al. Improvements in Between-Vendor MRI Harmonization of Renal T2 Mapping using Stimulated Echo Compensation. *J Magn Reson Imaging*. February 2024. doi:10.1002/jmri.29282

13. Horvat-Menih I, Li H, Priest AN, et al. High-resolution and highly accelerated MRI T2 mapping as a tool to characterise renal tumour subtypes and grades. *Eur Radiol Exp*. 2024;8:76. doi:10.1186/s41747-024-00476-8

14. Henry R, Goetsch T, Brandhuber L, et al. MRI quantitative T1 and T2 mapping of the renal cortex: Assessment of normal values and potential usefulness for renal masses at 3 T. *Eur J Radiol*. 2024;181:111741. doi:10.1016/j.ejrad.2024.111741

15. Wolf M, de Boer A, Sharma K, et al. Magnetic resonance imaging T1- and T2-mapping to assess renal structure and function: a systematic review and statement paper. *Nephrol Dial Transplant*. 2018;33(suppl_2):ii41-ii50. doi:10.1093/ndt/gfy198

Supplementary Information 1

To investigate the choice of the maximum TE on measured T_2_ for the SE-EPI sequence, a phantom data set was collected with TEs of 20 - 160 ms in 10 ms steps and the associated T_2_ maps were generated using all echo times up to a maximum TE from 30 - 160 ms (Figure S1). Reducing the maximum TE to <= 40 ms decreased the accuracy of the longer T_2_ spheres, even those within the physiological T_2_ kidney range. Increasing the maximum TE to >= 100 ms resulted in inaccurate quantification of the shortest T_2_ spheres as the exponential fit was then dominated by echoes close to the noise floor.


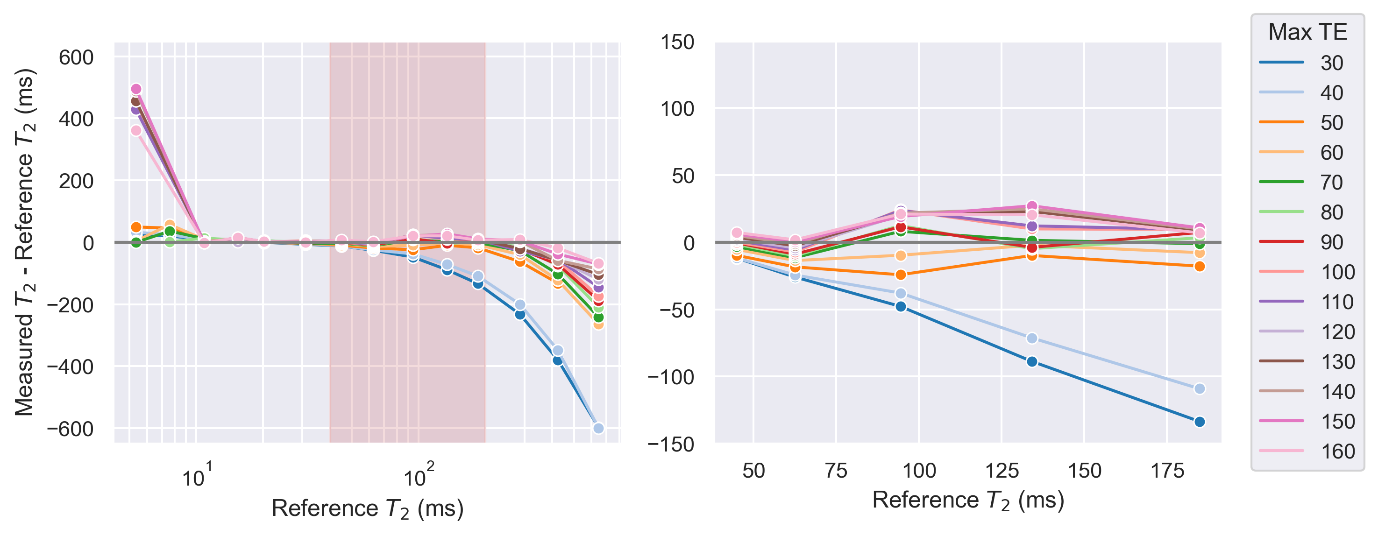


***Figure S1:*** *ISMRM/NIST phantom data acquired using the SE-EPI sequence with echo times from 20 – 160 ms in 10 ms steps. Data were fit with an increasing number of echo times up to a maximum TE of 30 – 160 ms to investigate the effect on measured T_2_. Left: Average measured T_2_ within each sphere is compared to reference T_2_ values on a logarithmic x-axis for the full range of T_2_ spheres with the physiological range of kidney T_2_ shaded in red. Right: Spheres with reference T_2_ values in the physiological range of kidney T_2_ shown on a linear x-axis.*

Supplementary Information 2

Signal intensity versus echo time plots as a function of B_1eff_ from 30 -100% of nominal flip angle. Note the reduction in accuracy on reducing B_1eff_, with all sequences being very inaccurate when B_1eff_ < 50% The shorter the T_2_ of a sphere the greater overestimation of measured T_2_ in these spheres. Note, a SE-EPI is expected to be invariant to the flip angle (i.e. 45^o^-90^o^ pairing would result in same measured T_2_ as 90^o^-180^o^ but with lower SNR). However, our vendor SE-EPI scheme applies a shortened RF pulse for the shortest SE-EPI echo time compared to later echo times, this leads to different sensitivity to B_1_ with TE and an increase in fitted T_2_ at lower flip angle, particularly for the shortest T_2_ spheres.

*
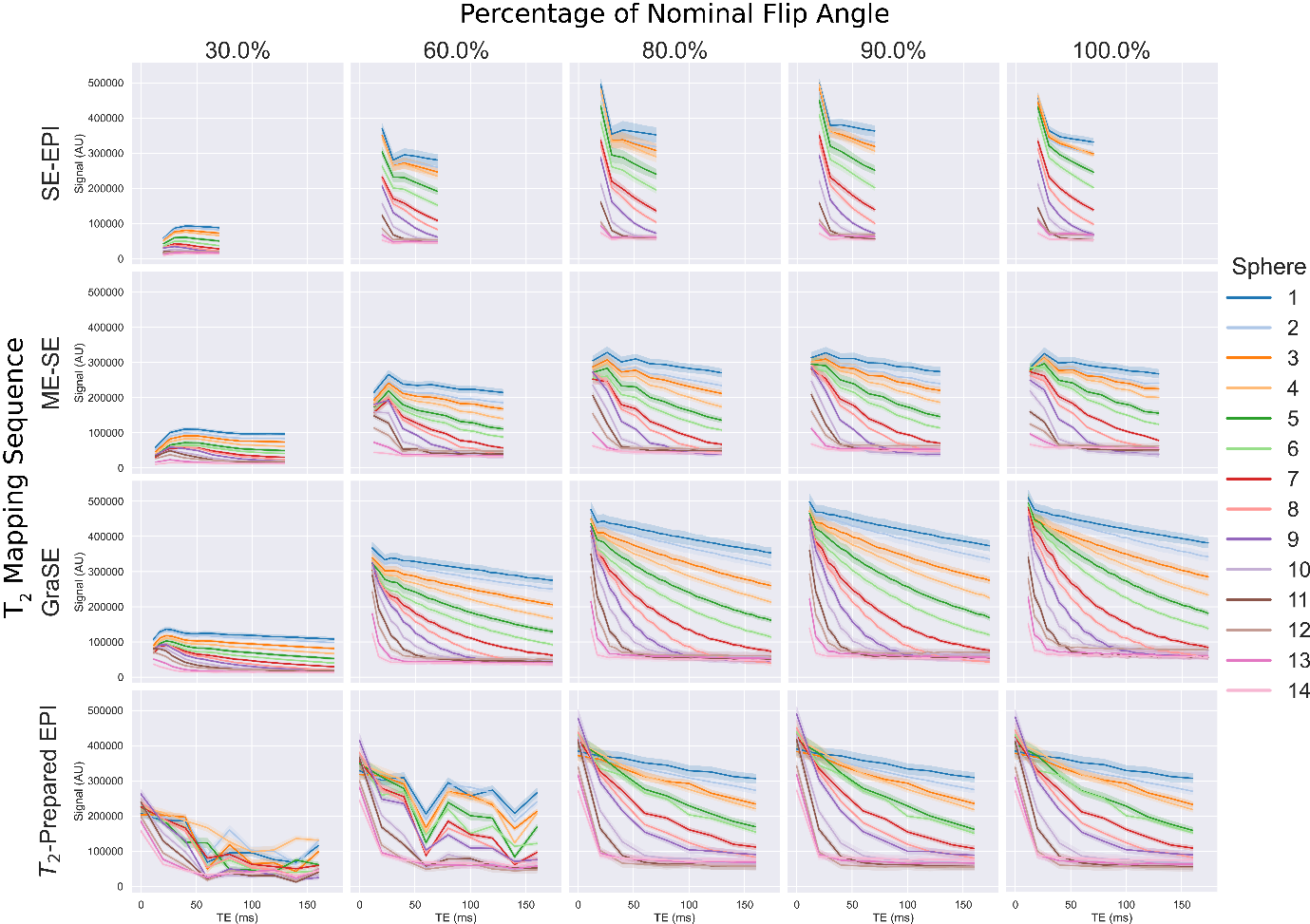
*

| **Sphere** | **Reference T2 (ms)** | **Sphere** | **Reference T2 (ms)** |
| --- | --- | --- | --- |
| 1 | 581.3 ± 0.4 | 8 | 46.42 ± 0.01 |
| 2 | 403.5 ± 0.6 | 9 | 31.97 ± 0.08 |
| 3 | 278.1 ± 0.3 | 10 | 22.56 ± 0.01 |
| 4 | 190.91 ± 0.01 | 11 | 15.813 ± 0.006 |
| 5 | 133.27 ± 0.07 | 12 | 11.237 ± 0.006 |
| 6 | 96.89 ± 0.05 | 13 | 7.911 ± 0.004 |
| 7 | 64.07 ± 0.03 | 14 | 5.592 ± 0.006 |

***Figure S2:*** *Signal intensity versus echo time (TE) for each sphere of the ISMRM/NIST phantom T_2_ array imaged for each of our T_2_ mapping sequences with different % of nominal flip angle (FA) to simulate a range of B_1eff_.*
